# Nerve repair with polylactic acid and inosine treatment enhance regeneration and improve functional recovery after sciatic nerve transection

**DOI:** 10.3389/fncel.2024.1525024

**Published:** 2025-01-06

**Authors:** Fellipe Soares dos Santos Cardoso, Guilherme dos Santos Maria, Fernanda Marques Pestana, Ricardo Cardoso, Bruna dos Santos Ramalho, Luiza dos Santos Heringer, Tiago Bastos Taboada, Ana Maria Blanco Martinez, Fernanda Martins de Almeida

**Affiliations:** ^1^Laboratório de Neurodegeneração e Reparo – Departamento de Anatomia Patológica, Hospital Universitário Clementino Fraga Filho, HUCFF/UFRJ, Rio de Janeiro, Brazil; ^2^Faculdade Souza Marques, Rio de Janeiro, Brazil; ^3^Departamento de Histologia ICB/UFRJ, Instituto de Ciências Biomédicas, Rio de Janeiro, Brazil

**Keywords:** nerve repair, inosine, sciatic nerve transection, nerve regeneration, A2A receptor

## Abstract

**Background:**

Following transection, nerve repair using the polylactic acid (PLA) conduit is an effective option. In addition, inosine treatment has shown potential to promote nerve regeneration. Therefore, this study aimed to investigate the regenerative potential of inosine after nerve transection and polylactic acid conduit repair.

**Methods:**

C57/Black6 mice were subjected to sciatic nerve transection, repair with PLA conduit, and intraperitoneal injection of saline or inosine 1 h after injury and daily for 1 week. To assess motor and sensory recovery, functional tests were performed before and weekly up to 8 weeks after injury. Following, to evaluate the promotion of regeneration and myelination, electroneuromyography, morphometric analysis and immunohistochemistry were then performed.

**Results:**

Our results showed that the inosine group had a greater number of myelinated nerve fibers (1,293 ± 85.49 vs. 817 ± 89.2), an increase in neurofilament high chain (NFH) and myelin basic protein (MBP) immunolabeling and a greater number of fibers within the ideal g-ratio (453.8 ± 45.24 vs. 336.6 ± 37.01). In addition, the inosine group presented a greater adenosine A2 receptor (A2AR) immunolabeling area. This resulted in greater compound muscle action potential amplitude and nerve conduction velocity, leading to preservation of muscle and neuromuscular junction integrity, and consequently, the recovery of motor and sensory function.

**Conclusion:**

Our findings suggest that inosine may enhance regeneration and improve both motor and sensory function recovery after nerve transection when repaired with a poly-lactic acid conduit. This advances the understanding of biomaterials and molecular treatments.

## 1 Introduction

Traumatic injuries to the sciatic nerve result in psychosocial burden, loss of function and neuropathic pain in the affected lower extremity, affecting quality of life and socioeconomic status (Deumens et al., 2010).

Following axotomy, a complex event called Wallerian degeneration occurs in the distal part of the nerve immediately after injury. Due to impaired axonal transport, levels of axoplasmic nicotinamide mononucleotide adenylyl transferase (NMNAT 2) are reduced and subsequent activation of sterile alpha and TIR motif containing 1 (SARM-1) causes a decrease in nicotinamide adenine dinucleotide (NAD+) levels, leading to energy failure and Ca + 2 release in the axoplasm. This in turn activates the calpains, leading to axonal collapse (Wang et al., 2012; Yang et al., 2015; Czech et al., 2023). A remarkable feature of peripheral nerve injury is the reprogramming of Schwann cells (SCs) into a repair state, with remarkable downregulation of myelin-associated genes and upregulation of the transcription factor c-Jun in myelinating SC (Arthur-Farraj et al., 2012; Gomez-Sanchez et al., 2017; Jessen and Mirsky, 2022). In addition, the Remak SC also reprograms into a repair state (Gomez-Sanchez et al., 2017). Thus, both repair SC acts conjointly and proliferate, initiate myelinophagy, recruit macrophages from the blood vessels to phagocytose axons and myelin debris and align within their basal lamina to form the Büngner bands (BB). This process creates a favorable environment and provides cues for regenerating axons to guide them through the distal stump to the target muscle, facilitating the formation of new synapses. Myelinating SCs then activate the remyelination program and Remak SCs form new Remak bundles (Allodi et al., 2012; Kim H. A. et al., 2013; Gomez-Sanchez et al., 2015; Boerboom et al., 2017).

Following nerve transection, nerve repair is necessary to promote axon regeneration (Chiono and Tonda-Turo, 2015). For small gaps (up to 1 cm), it is possible to connect the proximal and distal stumps directly. However, in gaps up to or higher than 5 cm, is necessary tension to coaptate the stumps, leading to regeneration impairment. Regarding this issue, grafting is required to avoid tension (Singh et al., 2022). In such cases, the preferred option is usually autograft, the gold standard technique for neurosurgeons. However, this approach leads to impaired sensitivity and neuropathic pain in the sural harvest area and could result in neuroma formation in the grafted area (Dalamagkas et al., 2016; Vijayavenkataraman, 2020). Nerve guide conduits (NGCs) have emerged as a promising solution to overcome these issues (Vijayavenkataraman, 2020). Among these, the PLA conduit is considered a viable option due to its low toxicity, high biocompatibility, and resorbable characteristics (Goulart et al., 2016; Domingues et al., 2017; Pestana et al., 2018; Durço et al., 2020).

Inosine, a purine nucleoside derived from the deamination of adenosine, is an agonist of the adenosine or P1 receptors and acts by binding to them (Welihinda et al., 2016; Borea et al., 2018). Inosine binds directly to the A2A receptor and activates adenylyl cyclase, increasing the expression of ciclic adenosine monophosphate (cAMP), which in turn phosphorylates cyclic AMP-dependent protein kinase (PKA), effectively phosphorylating the mammalian sterile 20-like kinase-3b (MST3b) protein and regulating the growth associated protein-43 (GAP 43) protein (Lorber et al., 2009; Qiu et al., 2023). In addition, A2A binding increases the expression of brain-derived neurotrophic factor (BDNF), promote the function of both BDNF and nerve growth factor (NGF) and transactivates the tyrosine kinase B (TrkB) receptor without BDNF binding (Diógenes et al., 2015; Ribeiro et al., 2016). Inosine also promotes neuroprotection through its breakdown product ribose-5-phosphate, which increases adenosine triphosphate (ATP) levels under stress conditions (Jurkowitz et al., 1998). Inosine treatment is widely used and has been described in the context of central nervous system (CNS) injury (Zai et al., 2011; Kim D. et al., 2013; Kuricova et al., 2014), but it can also be used following peripheral nerve injury (PNI).

Previously, we showed that animals treated with inosine after nerve crush had an abundance of motor and sensory neurons, myelinated fibers and blood vessels 2 weeks after injury. There were also fewer myelin sheaths and macrophages in the microenvironment. These findings ultimately contributed to the recovery of motor and sensory function (Cardoso et al., 2019). Although inosine is beneficial in a crush injury model, its effects on promoting regeneration after severe nerve injury are unknown. With this in mind, the main aim of this study is to investigate the regenerative potential of inosine following sciatic nerve transection and repair using a PLA conduit in mice.

## 2 Materials and methods

### 2.1 Experimental procedures

All experimental procedures were approved by the Ethical Committee for Animal Research of the Health Science Centre of the Federal University of Rio de Janeiro (protocol number CEUA 080/23). All animal handling and surgical procedures strictly followed the approved guidelines of the committee. Animals were obtained from the colony bred at BioRio (Rio de Janeiro, Brazil).

### 2.2 Surgical procedure

Young adult female C57/BL6 mice (8–12 weeks old) were anesthetized intraperitoneally with ketamine (100 mg/kg) and xylazine (15 mg/kg). An incision was made in the right hind limb to expose the sciatic nerve without the muscle segment. The sciatic nerve was then transected anterior to the bifurcation using an ophthalmic microscissor to create a gap and remove 3 mm of nerve. The nerve was then repaired using a 5 mm long conduit, with the proximal and distal stumps inserted and sutured to the epineurium with a 10.0 mono-nylon suture. After surgery, we replaced the muscles and sutured the skin with 6.0 nylon monofilament. In this study, we chose to use the PLA conduit, which presents several advantages to support regeneration as described by Goulart et al. (2016) and Pestana et al. (2018). The conduit was manufactured and supplied by the Macromolecules Institute of the Federal University of Rio de Janeiro, Brazil (IMA/UFRJ).

### 2.3 Treatment administration

One hour following nerve transection and repair with PLA conduit, we randomly administered, by intraperitoneal injection, saline or inosine (260 mM–70 mg/day/kg body weight, SIGMA) (Kim D. et al., 2013). Thus, the animal groups were designated saline + PLA and inosine + PLA. The treatment was carried out daily until 7 days after injury.

### 2.4 Perfusion and tissue harvesting

Eight weeks after injury, animals were anesthetized and transcardially perfused with 4% paraformaldehyde in 0.1 M phosphate buffer (pH 7.4). After perfusion, the animals were dissected (*n* = 5 per group) and the regenerated nerve and gastrocnemius muscle were harvested. The regenerated nerve was divided into proximal, middle, and distal segments. In addition, the medial region of the gastrocnemius muscle was cut transversely and divided into proximal and distal parts. The distal segment of the gastrocnemius muscle and the regenerated nerve were postfixed with 4% paraformaldehyde in 0.1 M phosphate buffer (pH 7.4). The middle segment of the regenerated nerve was postfixed in 2% glutaraldehyde in 0.1 M phosphate buffer (pH 7.4) for further analysis.

### 2.5 Light microscopy and transmission electron microscopy

After fixation by immersion in 2.5% glutaraldehyde, the middle segment of regenerated nerves was post-fixed in 1% osmium tetroxide containing 0.8% potassium ferrocyanide and 5 mM calcium chloride in 0.1 M cacodylate buffer (pH 7.4) for 1 h. Then, the segments were washed in 0.1 M cacodylate buffer (pH 7.4) and contrasted overnight in 1% aqueous uranyl acetate solution. Nerves were dehydrated in graded acetone, infiltrated with Embed-812 resin (Electron Microscopy Sciences) and polymerized at 60°C for 48 h. Semi-thin (500 ηm) and ultra-thin (70 ηm) cross-sections were obtained on an RMC MT-6000 ultramicrotome. The semi-thin sections were stained with 1% Toluidine Blue and analyzed under a light microscope (model Axioskop 2 Plus, Zeiss). In addition, the ultra-thin sections were collected on copper grids, and analyzed in the transmission electron microscope (model, EVO MA10, Zeiss).

### 2.6 Morphometric analysis

To assess regeneration and angiogenesis, images of the semi-thin sections were taken at 63x magnification using the Axiovision Rel. 4.5 program. The entire nerve was imaged and merged using Adobe Photoshop CS6. The total number of myelinated fibers and the total number of blood vessels were then counted using the plugin cell counter at ImageJ software (1.42q, USA).

Furthermore, five areas of the same semithin sections were obtained at 100x magnification to quantify fiber area, axon area, myelin area, and G ratio. The Axon area refers to the inner area of the axon, while the fiber area includes both the axon and the myelin sheath. Therefore, we subtract the axon area from the fiber area to determine the myelin area. For the g-ratio, we used the image J (1.42q, USA) and obtained values for the axon diameter and fiber diameter. Then, the g-ratio was determined by dividing the axon diameter by the fiber diameter and the results were stratified into ranges. According to Chomiak and Hu (2009), the optimal g-ratio range in the sciatic nerve is 0.55–0.68.

### 2.7 Muscle analysis

To evaluate muscle atrophy, we weighed the ipsilateral and contralateral gastrocnemius muscles before sectioning them for further analysis. After dissection and post-fixation as described in section 2.4, we dried both muscles using non-abrasive paper and weighed them on a precision balance. The weight of the dried muscles was measured in grams.

To investigate the number of neuromuscular junctions (NMJs), the distal section of the gastrocnemius muscle was stained with *α*-bungarotoxin. The tissue was fixed and divided following section 2.4. The distal section of the gastrocnemius was washed in 0.1 M PBS (pH 7.4), cryoprotected in 10, 20, and 30% PBS-sucrose, embedded in OCT (Tissue-Tek, Tokyo, Japan), and frozen. Sixty μm longitudinal frozen sections were collected on gelatin-coated glass slides using a cryostat (Leica CM 1850, Germany) and stored in a freezer. The slides were placed at room temperature and then washed in PBS and PBS 0.3% Triton. Then, slides were incubated in α-Bungarotoxin (conjugated with Alexa Fluor 594, 1:500; Molecular Probes, USA) for 2 h. Then, the slides were washed in PBS and finally mounted with Fluoromount (Sigma). To evaluate the number of NMJ, the slides were observed under a fluorescence microscope (model Axioskop 2 plus, Zeiss) and each NMJ was manually counted. Lastly, we photographed the slides under a confocal microscope (model Celldiscoverer 7, Zeiss).

### 2.8 Immunohistochemistry

The distal segment of the regenerated nerve was post-fixated and cryoprotected following topics 2.4 and 2.7.2, respectively. Ten μm frozen transverse serial sections of the distal segment of the regenerated nerve were made and collected on gelatin-coated glass slides. Eight weeks after nerve repair and inosine treatment we performed immunohistochemistry for NFH, a neurofilament marker, A2AR, a P1 adenosine receptor marker, and MBP, a myelin marker. Slides were washed in phosphate buffered saline (PBS), incubated in blocking solution (10% normal goat serum in PBS 0.1 M and 0.3% Triton) for 1 h, washed in PBS 0.3% triton, and then incubated overnight in primary antibody mouse anti-NFH (1:1000; Sigma), rabbit anti-A2AR (1:200; Invitrogen) and rat anti-MBP (1:200; Millipore). Sections were washed in PBS-triton 0.3%, incubated in the secondary antibody Alexa 488 goat anti-mouse (1:500; Invitrogen), Alexa 488 goat anti-rat (1:500; Invitrogen) or Alexa 546 goat anti-rabbit (1:500; Invitrogen) for 1:30 h. Then sections were washed in PBS-triton 0.3%, nuclei were stained with 4,6-diamino-2-fenilindol (DAPI) (1:10,000; Molecular Probes, USA), washed in PBS and distilled water, and finally mounted with Fluoromount (Sigma).

Primary antibodies were omitted for all negative controls. Images were acquired using a confocal microscope (model Celldiscoverer 7, Zeiss), and the immunolabeled area was quantified using the Image-Pro Plus software, employing the measurement parameter per area, which considers the ratio of area of the object/total area of an image.

### 2.9 Functional analysis

Motor function was evaluated using the SFI, based on a protocol described by Inserra and collaborators (1998). The animal’s paw prints were registered weekly up to 8 weeks and two measurements were taken: the print length (PL), corresponding to the distance from the heel to the third toe, and the toe spread (TS), corresponding to the distance from the first to the fifth toe. To determine the significant contribution to the index formula, the TS and PL from the normal side (NTS and NPL) were subtracted from the experimental side (ETS and EPL). Thus, the SFI was calculated according to the following equation:


$$SFI=118.9\times \left(\frac{ETS-NTS}{NTS}\right)-51.2\times \left(\frac{EPL-NPL}{NPL}\right)-7.5$$

A normal SFI around zero corresponds to normal nerve function and an SFI around – 100 represents total loss of sciatic nerve function.

For the pinprick test, the animals were placed in acrylic boxes, whose floor is a mesh of 5 mm^2^ made of non-malleable 1-mm-thick wire, for 15 min before the experiment to acclimate to the environment. Mirrors were positioned F25 cm below the experimental box to facilitate visualization of the mouse paw plant surface. After acclimation, an entomological pin (0.25 mm diameter entomological pin-Papillon) was gently applied to the lateral plantar surface of the hind paws without moving the paws or penetrating the skin of the animal. The hind paws were divided from the heel to the most lateral toe. The pin was applied twice in each area and a response was considered positive (1) when the animal removed or flicked its paw and zero when no response was observed (Ma et al., 2011).

For the analgesimeter, animals were placed in the same acrylic boxes described above. The paw-withdrawal threshold, in grams, was evaluated weekly by inducing gradual pressure on the plantar surface of the hind paw, using the digital analgesimeter (electronic von Frey analgesimeter, Insight Equipment Ltda., Ribeirão Preto, SP, Brazil). Three measurements were taken and a mean was obtained.

### 2.10 Electroneuromyography

To assess the regeneration 8 weeks after nerve repair (saline + PLA and inosine + PLA *n* = 5; normal *n* = 3), animals were anesthetized with ketamine (100 mg/kg) and xylazine (15 mg/kg) and then the repaired sciatic nerve was exposed, as well as the triceps sural muscle and the calcaneus tendon. Using the Power Lab 4/35 device (ADInstruments, Germany), the electrical stimulus with the intensity of 10 mV was triggered both before and after the conduit through a hooked bipolar electrode, with the cathode distant 2 mm from the anode, and the active electrode was inserted into the triceps sural muscle while the reference electrode was positioned over its tendon. The ground electrode was placed under the skin at the base of the animal’s tail. To analyze the amplitude of the CMAP, we considered the trace recorded before the conduit. To analyze the latency, we recorded traces before and after the conduit and obtained the moment in milliseconds that the CMAP was triggered. To analyze the nerve conduction velocity (NCV), we considered the total distance of the conduit (5 mm or 0.005 m) and divided it by the difference of the latencies before and after the conduit, using the following equation:


$$NCV=\frac{\mathrm{\Delta distance}\left(m\right)}{\mathrm{\Delta latency}\left(s\right)}$$

The NCV was considered in m/s.

### 2.11 Statistical analysis

All statistical analyses were performed using Graph Pad Prism 8.0 (Graph Pad software, USA) and presented as mean ± standard *error of the mean*). We performed the t-test parametric (Welch correction) or non-parametric (Mann Whitney’s), depending on the normality test obtained. To analyze data with 3 or more groups, we used the one-way ANOVA, reported as one-tailed and Tukey’s.

## 3 Results

### 3.1 Inosine promotes regeneration after sciatic nerve transection and repair with PLA conduit

**Figure 1:**
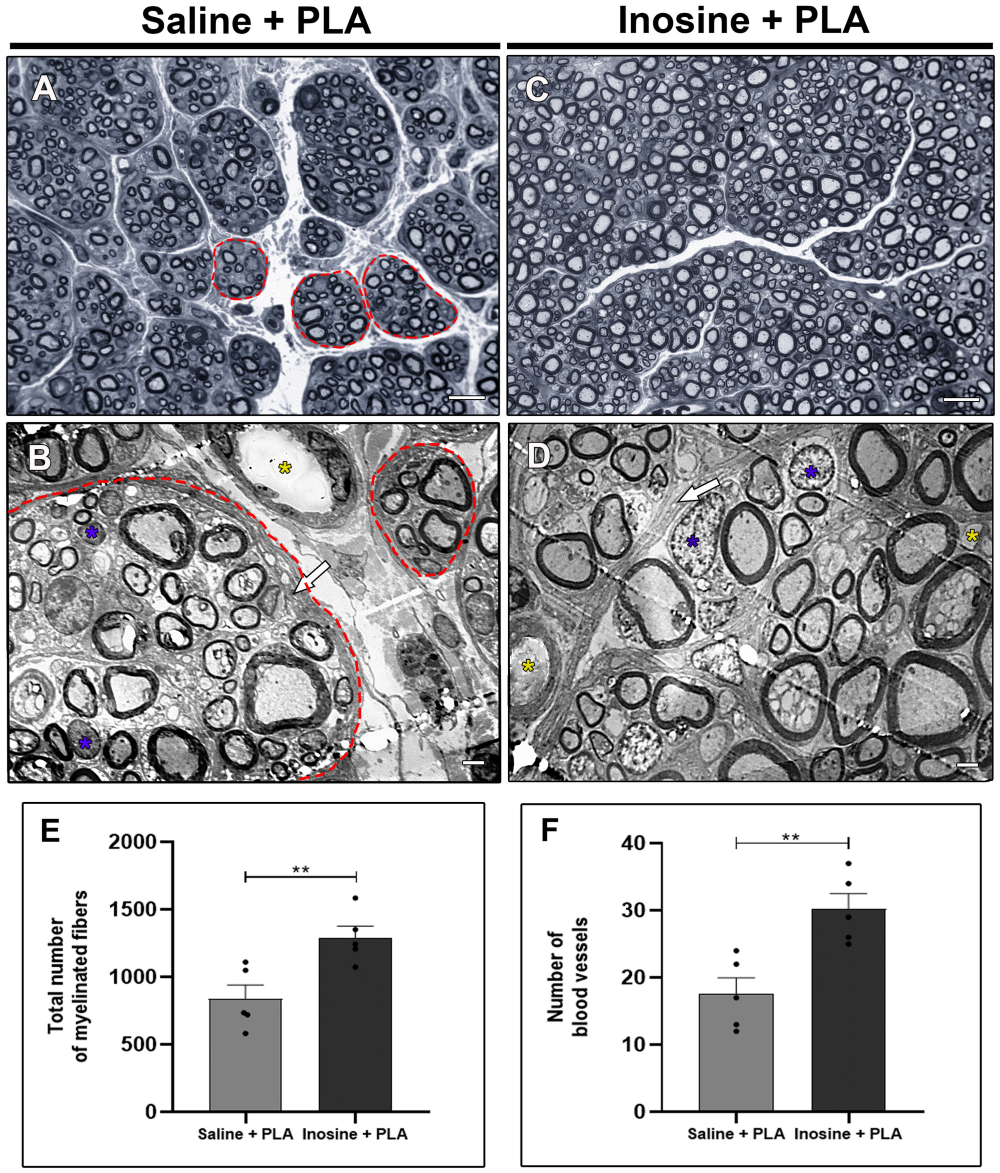
Semithin and ultrathin sections and quantitative analysis. Semithin section stained with Toluidine blue of saline + PLA group **(A)** and from inosine + PLA group **(C)**, and ultrathin section of saline + PLA **(B)** and inosine + PLA **(D)**. Note that saline + PLA has regenerative islets (red dot circles), different from inosine + PLA, which are surrounded by concentric perineurium layers (white arrow). In addition, the following graphics demonstrate the total number of myelinated fibers **(E)** and total number of blood vessels **(F)**. In both groups, blue asterisks represent Schwann cells. Scale bar = 10 μm **(A,C)** and 2 μm **(B,D)**; saline + PLA (*n* = 5) and inosine + PLA (*n* = 5); Values represent mean ± SEM (**p < 0.01).

To assess the regenerative potential of inosine 8 weeks after nerve injury and repair with a PLA conduit, we prepared toluidine blue-stained semi-thin sections and ultrathin sections from segment B of the regenerated nerve for qualitative and quantitative analysis in the saline + PLA (Figures 1A,B) and inosine + PLA (Figures 1C,D) groups.

First, we observed that the PLA conduit effectively guided the fibers in the right direction in both groups (Figures 1A,C). However, in the saline + PLA group, nerve fibers were clustered, as typically observed during the regeneration process after repair with a conduit, containing myelinated fibers, associated with SC (blue asterisk), which are surrounded by perineurium (Figures 1A,B; red dots). However, the inosine + PLA groups fibers are uniformly organized, also associated with SC (blue asterisk), nevertheless surrounded by compact perineurium layers (white arrow), allowing a better tissue organization (Figures 1C,D). Moreover, compared to the saline + PLA group, the inosine + PLA group presented a higher number of myelinated fibers (Figure 1E) (1,293 ± 85.49 vs. 817 ± 89.2; **p < 0.01), and also showed a higher number of blood vessels (Figure 1F) (30.2 ± 23 vs. 17.20 ± 2.7; **p < 001), demonstrating that inosine can stimulate both the nerve regenerative potential and angiogenesis.

**Figure 2:**
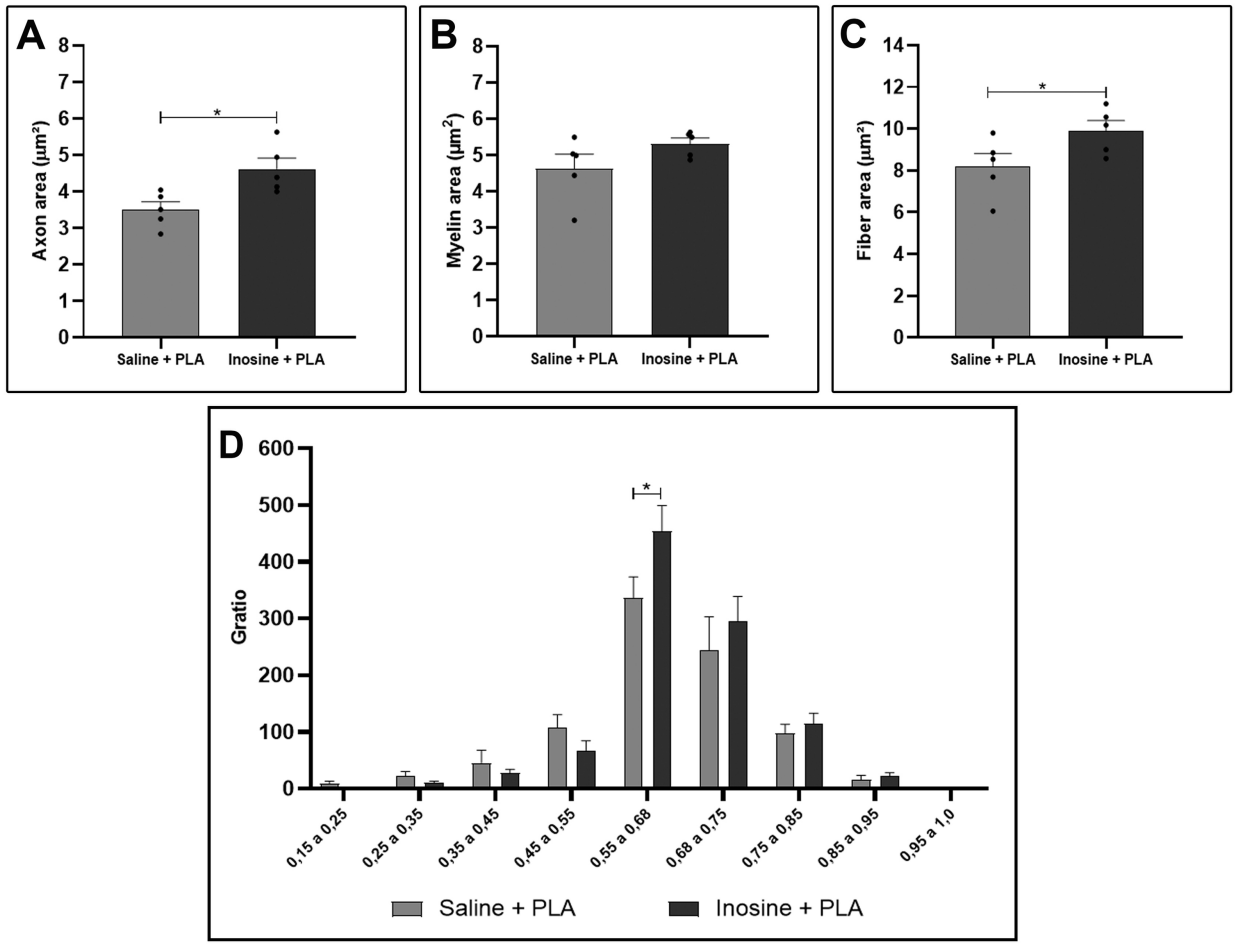
Morphometric analysis of myelinated nerve fibers in semithin sections. Quantitative analysis of axon area **(A)**, myelin area **(B)**, fiber area **(C)**, and g-ratio **(D)** from saline + PLA and inosine + PLA groups. Saline + PLA (*n* = 5) and inosine + PLA (*n* = 5); Values represent mean ± SEM (*p < 0.05).

For the morphometric analysis, we evaluated the axon, myelin, and fiber area, as well as the g-ratio. Regarding the axon area (Figure 2A) and the fiber area (Figure 2C), we observed that the inosine + PLA group had a significantly larger area compared to the saline + PLA group (4.62 ± 0.3 vs. 3.5 ± 0.22; 9.9 ± 0.49 vs. 8.2 ± 0.63, *p < 0.05 for both). However, no significant differences were observed in myelin area (Figure 2B). Concerning the g-ratio analysis (Figure 2D), the inosine + PLA group exhibited a greater number of myelinated fibers in the ideal range of 0.55 to 0.68 (Chomiak and Hu, 2009) compared to the saline + PLA group (453.8 ± 45.24 vs. 336.6 ± 37.01, *p < 0.05). This result demonstrates the positive impact of the PLA tube and inosine treatment on remyelination after the transection injury.

**Figure 3:**
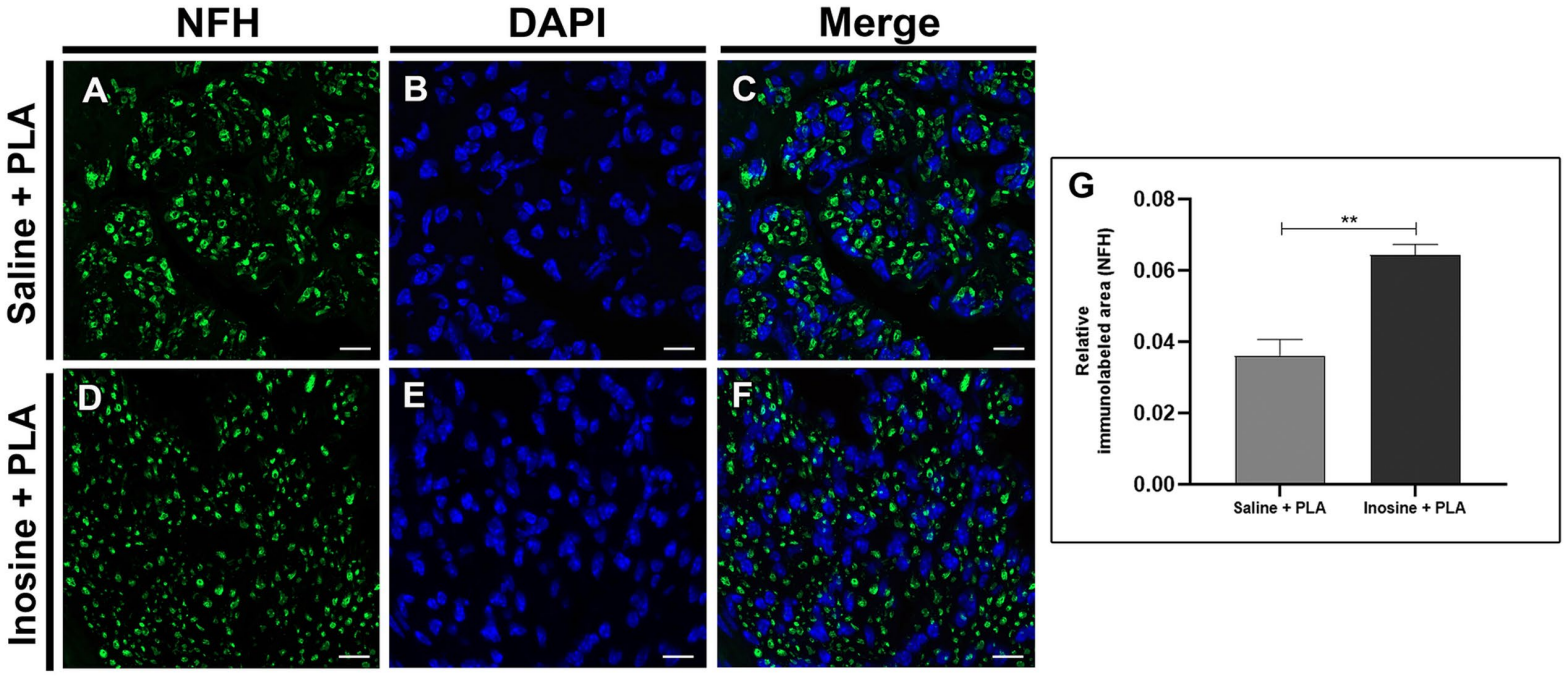
Relative immunolabeled area for NFH. Cross-section of sciatic nerve labeled for NFH (green), DAPI (blue), and merge of saline + PLA **(A–C)** and inosine + PLA **(D–F)**, and the graphic of relative immunolabeled area **(G)**. Scale bar = 25 μm; Saline + PLA (*n* = 3) and inosine + PLA (*n* = 3); Values represent mean ± SEM (**p < 0.01).

We also evaluated regeneration, performing immunohistochemistry for NFH (Figure 3). Regarding the relative immunolabeled area for NFH (Figure 3G), the inosine + PLA group (0.065 ± 0.003) (Figures 3D–F) showed a significantly larger immunolabeled area compared to the saline + PLA group (0.037 ± 0.004; **p < 0.01) (Figures 3A–C). This result further supports the potential of inosine in promoting nerve regeneration and repair when combined with a PLA tube after nerve injury.

### 3.2 Inosine enhances A2AR expression and myelination after nerve transection and repair with PLA conduit

To determine the presence of the A2A receptor in the sciatic nerves and its potential presence in Schwann cells, we performed an immunohistochemistry for the A2A receptor and the myelin basic protein (MBP), 8 weeks after the injury.

**Figure 4:**
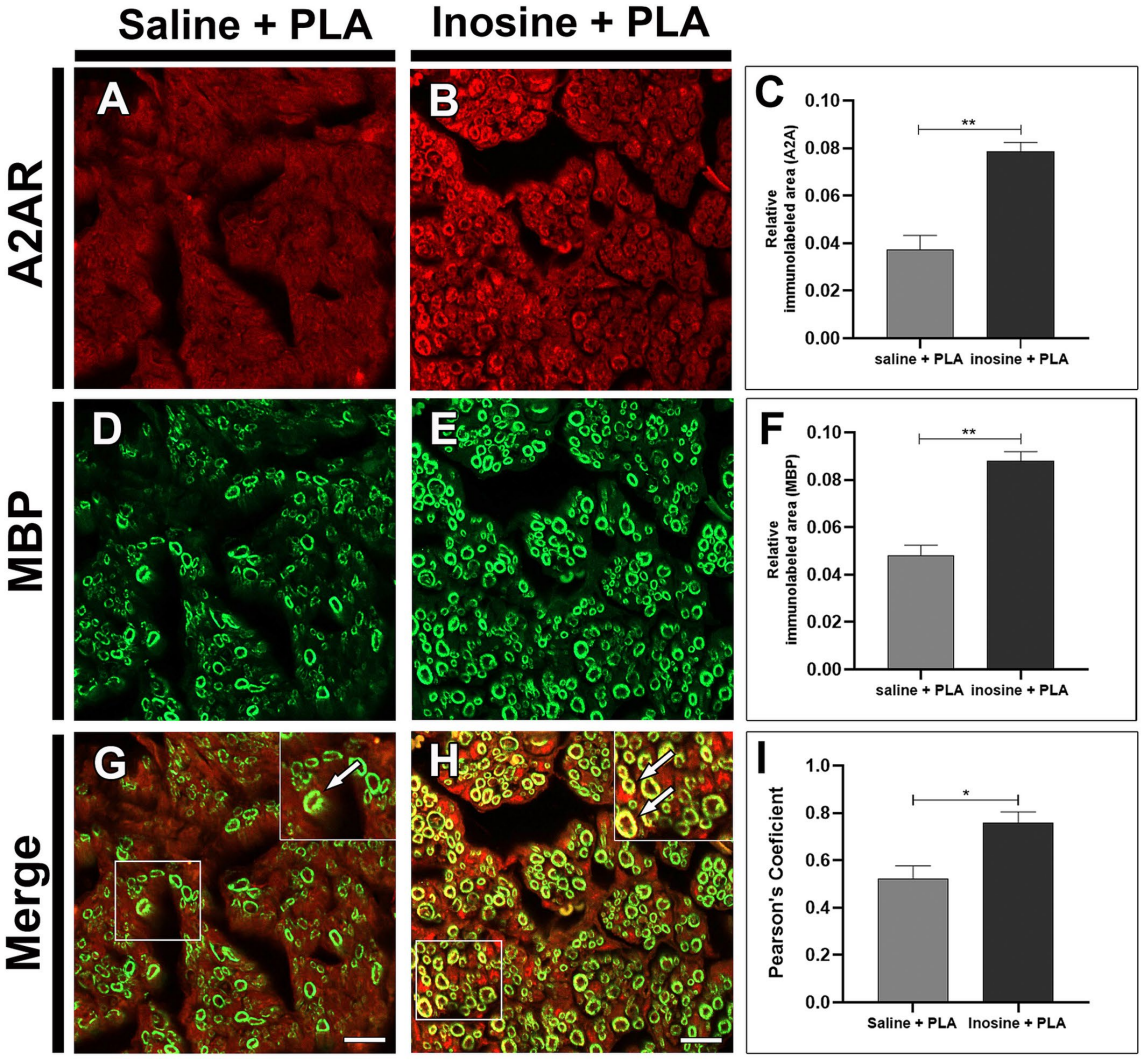
Relative immunolabeled area for A2A and MBP. Cross-section of sciatic nerve labeled for A2A, MBP, and merge, from saline + PLA **(A,D,G)** and inosine + PLA animals **(B,E,H)**, and graphics for the immunolabeled area of A2A (red) **(C)** and MBP (green) **(F)**. In addition, the quantification of the colocalization between A2AR and MBP (**G,H**, white arrows), are demonstrated by Pearson’s coefficient in graph **(I)**. Scale bar = 25 μm; Saline + PLA (*n* = 3) and inosne + PLA (*n* = 3); Values represent mean ± SEM (*p < 0.05 and **p < 0.01).

After injury, the A2A receptors are present in both groups (Figures 4A,B). However, the inosine + PLA group exhibited a greater immunolabeling area (0.078 ± 0.0037) compared to the saline + PLA group (0.037 ± 0.0059; **p < 0.01) (Figure 4C). This suggests that the treatment influences A2A receptor expression. Then, the MBP labeling was assessed (Figures 4D,E), and the inosine + PLA presented a larger immunolabeled area (0.088 ± 0.0039) compared to the saline + PLA group (0.048 ± 0.0042; **p < 0.01) (Figure 4F), consistent with the total number of myelinated fibers previously demonstrated in the inosine + PLA group.

The pattern of immunolabeling for A2A receptor resembles the myelin MBP immunolabeling. To investigate this, the colocalization between A2A and MBP (Figures 4G,H; white arrows in inserts) was assessed using Pearson’s analysis (Figure 4I). This analysis demonstrated a strong tendency for A2A/MBP colocalization in the inosine + PLA group (0.750 ± 0.046) compared to the saline + PLA group (0.512 ± 0.055; *p < 0.05).

### 3.3 Inosine attenuates muscle atrophy and preserves NMJ after nerve transection and repair with PLA conduit

After an injury, muscle atrophy emerges as a complication, impairing reinnervation. Regarding this issue, we evaluated muscle atrophy using the dry weight and the number of NMJs in the gastrocnemius muscle.

**Figure 5:**
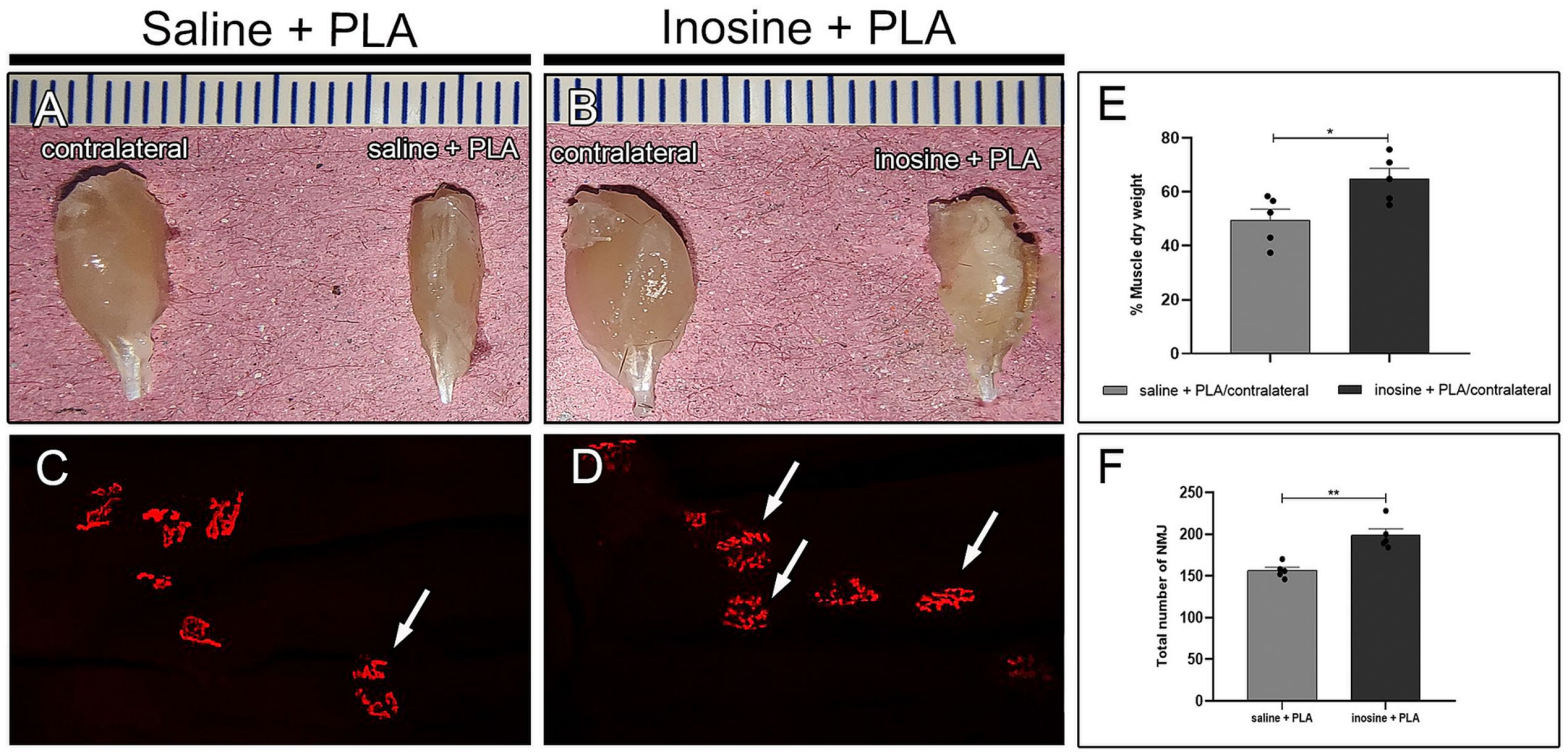
Muscle weight and NMJ assessment. Dissected gastrocnemius muscle from contralateral and saline + PLA **(A)** and contralateral and inosine + PLA **(B)**. The quantification of dry weight is demonstrated in graph **(E)**. The αbungarotoxin (red) labeling for saline + PLA **(C)** and inosine + PLA **(D)**. The graphic demonstrates the quantification of NMJ **(F)**. Moreover, note the presence of “pretzel” shape NMJ (white arrows), in both groups. Scale bar = 25 μm; Saline + PLA (*n* = 5) and inosine + PLA (*n* = 5); Values represent mean ± SEM (*p < 0.05 and **p < 0.01).

To assess muscle mass loss, the gastrocnemius from the contralateral and experimental sides were dried and weighed (Figures 5A,B), and a ratio (%) between the experimental and contralateral was obtained (Figure 5E), which in inosine + PLA is significantly higher compared to saline + PLA (64.8 ± 3.87 vs. 49.6 ± 4.03; *p < 0.05). Then, to assess the quantity of NMJs in the reinnervated muscles of animals subjected to repair with a PLA tube and inosine treatment, we used the *α*-bungarotoxin labeling (Figures 5C,D), and quantified the total number of NMJs (Figure 5F). The inosine + PLA group had a significantly higher number compared to the saline group (199 ± 7.79 vs. 156 ± 3.97; **p < 0.01). Note that in both groups, the typical “pretzel” shape was visible (Figures 5C,D, white arrow).

### 3.4 Inosine improves motor and sensitive functions after nerve transection and repair with PLA conduit

**Figure 6:**
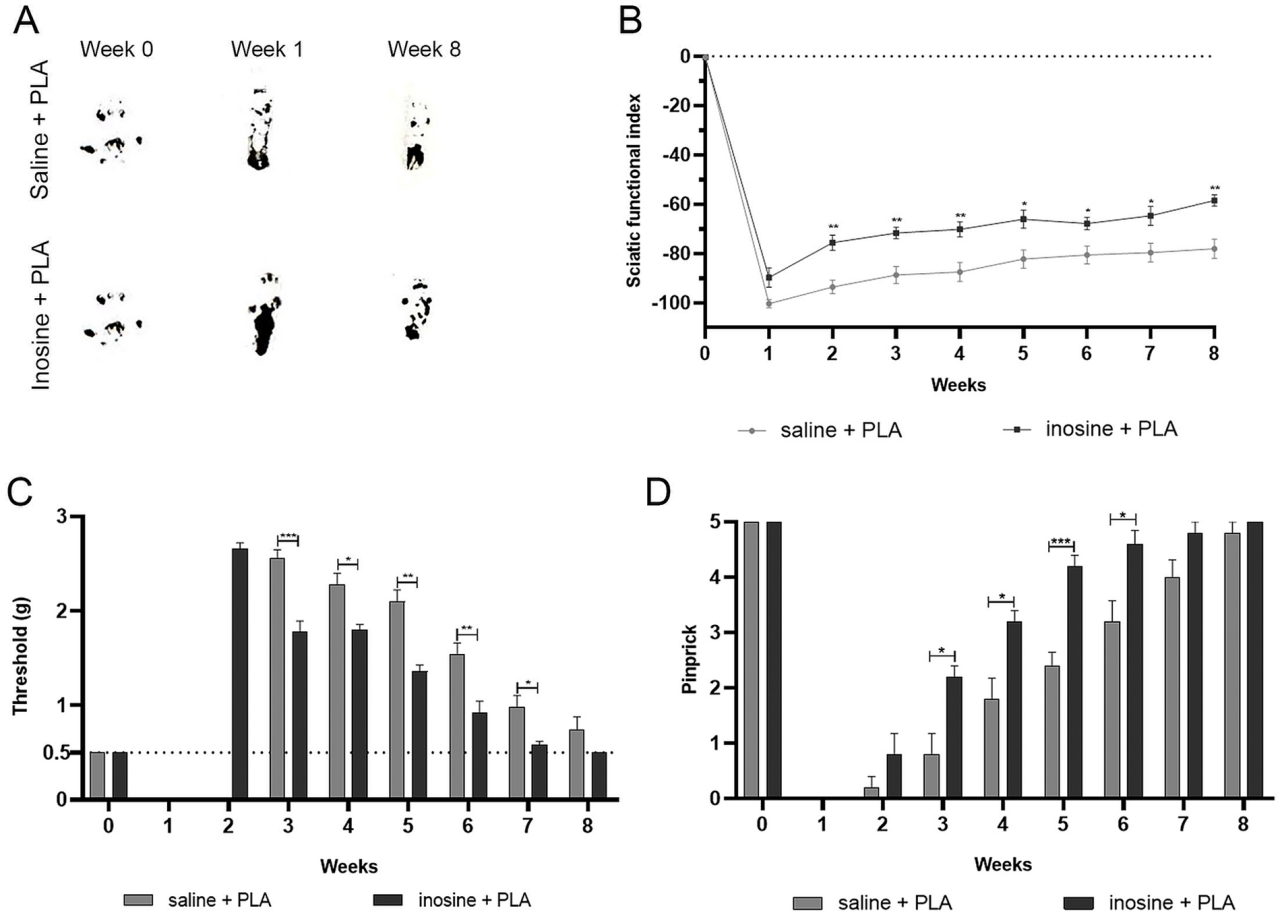
Functional analysis for motor and sensitive functions. To assess the motor function recovery, we performed the SFI. Footprints before (week 0) and after the injury (1 and 8 weeks) **(A)**. Note that inosine + PLA presented a better recovery in the footprints, reflecting in better SFI, as observed in graph **(B)**. To evaluate the recovery of sensitive functions, we performed analgesimeter **(C)** and pinprick **(D)**. Note that inosine + PLA group presented a complete recovery after 8 weeks, compared to saline + PLA. Saline + PLA (*n* = 5) and inosine + PLA (*n* = 5); Values represent mean ± SEM (*p < 0.05, **p < 0.01, and ***p < 0.001).

To evaluate the functional recovery 8 weeks after nerve repair and inosine treatment, we conducted functional assessment, using the SFI (Figures 6A,B), for motor recovery, and analgesiometer (Figure 6C) and pinprick test (Figure 6D) for sensitive recovery.

For motor function recovery, footprints were obtained, measured, and analyzed using the SFI formula (Inserra et al., 1998). The inosine + PLA group showed improvement in paw markers in the eighth week compared to the saline + PLA group (Figure 6A). As shown in the graph (Figure 6B), the inosine + PLA group demonstrated improved motor function recovery starting from the second week compared to the saline + PLA group (−75.59 ± 3.06 vs. −93.65 ± 2.72; *p < 0.05), maintaining until the eighth week (−58.49 ± 2.24 vs. −78.04 ± 3.82; **p < 0.01). However, it’s important to note that the inosine + PLA treatment did not result in a return to normal SFI levels.

Then, to assess tactile and pain sensitivity recovery, we performed the analgesiometer (Figure 6C) and pinprick tests (Figure 6D). In the first and second weeks, both groups showed complete loss of tactile sensitivity using the analgesiometer. However, from the third week onwards, significant recovery of tactile sensitivity in the inosine + PLA group compared to the saline + PLA group (1.78 ± 0.111 vs. 2.56 ± 0.08; ***p < 0.001) was observed. This higher tactile sensitivity recovery in the inosine + PLA group persisted until the seventh week (0.58 ± 0.03 vs. 0.98 ± 0.12; *p < 0.01) (Figure 6C). By the eighth week, there were no significant differences between the groups. Regarding the pinprick test, both groups had a total loss of pain sensitivity in the first week after the injury. By the second week, some animals in both groups began showing a response to pain, but there were no significant differences between the groups. However, from the third week onwards, the inosine + PLA group exhibited significant recovery of pain sensitivity compared to the saline + PLA group (2.2 ± 0.2 vs. 0.8 ± 0.37; ***p < 0.001). This recovery in pain sensitivity continued until the sixth week (4.6 ± 0.24 vs. 3.2 ± 0.37; *p < 0.001), after which no significant differences were observed between the groups (Figure 6D). Importantly, all animals in the inosine + PLA group achieved full recovery of function by the eighth week, which was not observed in the saline group.

### 3.5 Inosine enhances amplitude of CMAP and the NCV and decreases latency after nerve transection and repair with PLA conduit

**Figure 7:**
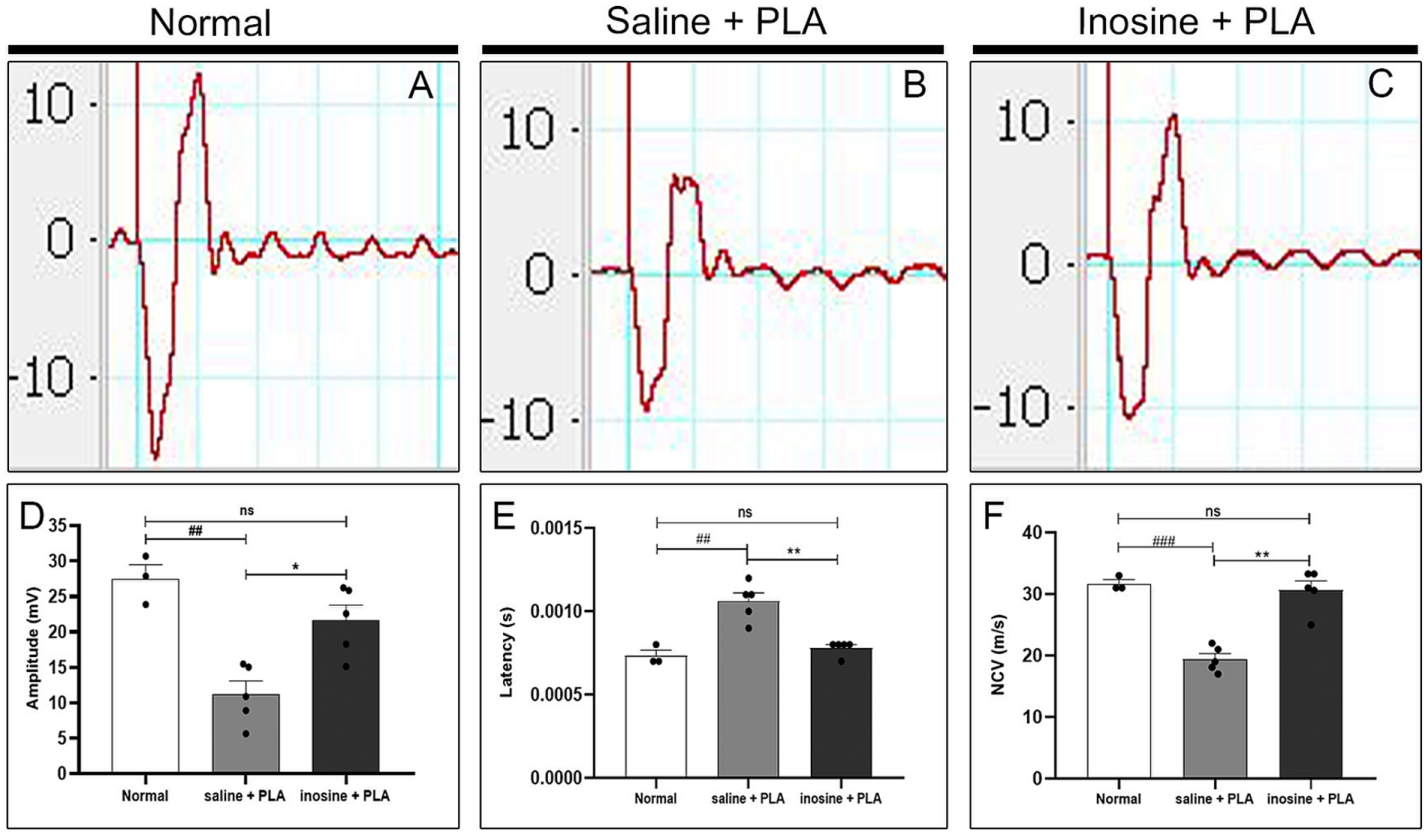
Electroneuromyography evaluation of amplitude and latency of CMAP and NCV. Traces of normal **(A)**, saline + PLA **(B)**, and inosine + PLA **(C)**, 8 weeks after nerve repair and treatment. To analyze the regeneration, we assessed the amplitude of CMAP **(D)** and to analyze the myelination, we assessed the latency of CMAP **(E)** and NCV **(F)**. Normal (*n* = 3); saline + PLA (*n* = 5) and inosine + PLA (*n* = 5); Values represent mean ± SEM (** and ## *p* < 0.01; *** and ### *p* < 0.001. # represents normal vs saline; * represents inosine vs saline).

To evaluate the implications of regeneration and remyelination 8 weeks after nerve repair and inosine treatment, we analyzed the amplitude and latency of CMAP and the NCV. The analysis of the CMAP traces demonstrates an apparent reduction in the amplitude in the saline + PLA group, compared with the normal and inosine + PLA (Figures 7A–C). The graph (Figure 7D) demonstrates that the amplitude in the normal group is significantly higher compared to the saline + PLA group (27.48 ± 1.97 vs. 11.96 ± 1.7; ^##^*p* < 0.01), and no difference was observed, comparing the normal with inosine + PLA groups (27.48 ± 1.97 vs. 21.20 ± 1.81; ns). However, the inosine + PLA group presents a higher amplitude of the CMAP compared to the saline + PLA group (21.20 ± 1.81 vs. 11.96 ± 1.7; *p < 0.05). This data confirmed the robust regeneration observed after inosine treatment.

Next, the latency of CMAP and nerve conduction velocity were evaluated. Regarding the latency of CMAP, the graph (Figure 7E) shows that in the saline + PLA group presented a higher latency compared to the normal group (1 × 10^−2^ ± 5 × 10^−5^ vs. 7.3 × 10^−3^ ± 3.3 × 10^−5^; ^##^*p* < 0.01), whereas there is no significant difference between the inosine + PLA group and normal group (7.8 × 10^−3^ ± 2 × 10^−5^ vs. 7.3 × 10^−3^ ± 3.3 × 10–^5^; ns). On the other side, the inosine + PLA group presented a significantly reduced latency compared to saline + PLA group (7.8 × 10^−3^ ± 2 × 10^−5^ vs. 1 × 10–^2^ ± 5 × 10^−5^; **p < 0.01). A similar pattern is observed for NCV (Figure 7F), where no difference was observed between the normal and inosine + PLA groups (31.67 ± 0.66 vs. 30.64 ± 1.51; ns), whereas significant differences are found between the normal and saline + PLA groups (31.67 ± 0.66 vs. 19.42 ± 0.92; ^###^*p* < 0.001), and between the inosine + PLA and saline + PLA groups (30.64 ± 1.51 vs. 19.42 ± 0.92; **p < 0.01). This data demonstrates effective myelination after nerve repair and inosine treatment.

## 4 Discussion

This study aimed to assess the potential of inosine treatment in promoting nerve regeneration after sciatic nerve transection and repair using a PLA conduit to bridge a 3 mm gap. The animals were treated with either saline or inosine, starting 1 h after the injury and continuing daily up to the first week, through intraperitoneal injection. After 8 weeks of functional analysis, inosine + PLA group presented significantly better recovery of both motor and sensory functions compared to the saline + PLA group. Additionally, inosine + PLA showed a closer approximation to the normal CMAP amplitude, reduced latency, and increased nerve conduction velocity. The improvements observed in the inosine + PLA group were attributed to a more organized microenvironment, a greater quantity of myelinated fibers, and more efficient myelination. In addition, we observed an increase in A2A receptor after treatment, suggesting an influence of this receptor after injury.

The use of nerve conduits to repair nerves after transection offers a significant advantage by providing physical support to guide regenerating fibers to the target organ. The PLA offers several advantages that support regeneration as previously described by Goulart et al. (2016) and Pestana et al. (2018). Our data show that the PLA tube guided regenerating fibers, as we observed regeneration clusters in the saline + PLA group, confirming previous studies (Goulart et al., 2016; Pestana et al., 2018). In contrast, the combination of inosine and PLA, besides guiding regenerated axons, resulted in a uniform distribution of regenerated fibers, different from the regenerative islands observed in the saline + PLA group, and also presented a compact perineurium layer, which in turn resulted in a better organization.

Previous studies demonstrated the efficacy of inosine treatment, both in severe models of injury in the CNS, such as in mild PNS injuries. After nerve transection, a severe nerve injury, inosine promoted a robust regeneration and angiogenesis, as inosine + PLA group presents a higher number of myelinated fibers, NFH immunolabeling, and blood vessels, corroborating with our previous data in nerve crush injuries (Cardoso et al., 2019) and also with severe models of SNC injuries (Chen et al., 2002; Kim D. et al., 2013). Additionally, we observed higher axon and fiber areas, resulting in a greater amount of fibers within the ideal g-ratio (0.55 and 0.68) (Chomiak and Hu, 2009). We also observed a larger immunolabeled area for MBP, suggesting an influence of inosine in myelination after nerve repair.

The A2A receptors are present in Schwann cells (Stevens et al., 2004). During development, pre-myelinating SC stimulated by adenosine or agonist of A2A receptor induces myelination (Fields, 2006). However, the mechanisms by which inosine, an agonist of A2A receptor (Welihinda et al., 2016), influences myelination following injury remain unknown. Injury or stress situations elevate the A2A receptor levels (Welihinda et al., 2016). Also, we injected inosine intraperitoneally. Thus, we considered that both situations may influence the higher immunolabeled area for A2A receptors in inosine + PLA animals we observed. In addition, we observed a strong colocalization between A2A receptor and MBP, indicating the presence of this receptor in SC. We observed a larger immunolabeling area for MBP in inosine + PLA, corroborating with the higher total number of myelinated fibers. Thus, we hypothesized that the inosine treatment by enhancing the A2A receptor, influenced myelination after injury. Fibroblasts, which are present in the perineurium, are responsible for forming the extracellular matrix and, have been found to express the A2A receptor on their surface (Shaikh and Cronstein, 2016), suggesting that they may react to inosine treatment, thereby contributing to the compaction of perineurium layers, and consequently, better organization observed in the ultrastructural analysis.

It is well-established that the longer the muscle denervation period following nerve injury, the greater the degree of muscle atrophy and consequent loss of NMJ viability due to their degeneration (Sulaiman and Gordon, 2000; Li et al., 2020). Our findings show that inosine enhanced nerve regeneration after repair. Specifically, we found that animals treated with inosine and PLA had a greater dry weight of the gastrocnemius muscle and a higher quantity of neuromuscular junctions (NMJs) compared to those treated with saline. This indicates that inosine helped prevent muscle atrophy and preserved NMJs, ultimately reducing muscle inactivity. These findings corroborate with those observed by Li and coworkers, regarding muscle denervation and regeneration (Li et al., 2020).

Regarding the functional analysis, we demonstrated that the inosine + PLA group partially recovered the motor function and completely the sensitive function. These findings are consistent with the observed significant better regeneration and attenuation of muscle atrophy. The partial recovery of motor function can be attributed to the severity of the injury. Furthermore, our findings align with previous findings on functional recovery following inosine treatment related to nerve crush injury (Cardoso et al., 2019) and central nervous system (CNS) injuries (Zai et al., 2011; Kim D. et al., 2013; Kuricova et al., 2014), thus corroborating with our findings.

The results from the ENMG show that the inosine + PLA group had a greater compound muscle action potential (CMAP) amplitude, latency, and nerve conduction velocity compared to the saline + PLA group. These values were similar to those observed in uninjured animals, which is a significant finding. This supports previous observations from both the total count of myelinated fibers, confirming the role of inosine in promoting regeneration, as demonstrated previously both in following CNS and PNS injuries (Benowitz et al., 1999; Chen et al., 2002; Kim D. et al., 2013; Cardoso et al., 2019). Furthermore, NCV relies on correct and robust myelination during development. Thus, considering that the NCV was higher in the inosine + PLA group when compared to the saline + PLA group, it supports the hypothesis that inosine influences myelination through A2A receptor activation in SC after injury. Nonetheless, more research is necessary to confirm this hypothesis.

## 5 Conclusion

Our research showed that the association of nerve repair with PLA conduit with inosine treatment promotes robust regeneration and stimulates angiogenesis. This leads to a reduction in muscle atrophy and a preservation of NMJ. Additionally, there is an improved organization of the microenvironment and better myelination of nerve fibers. The ENMG supports greater regeneration and also efficient remyelination in the inosine group, indicating an improved recovery of motor and sensory functions in the animals. We observed an increase in A2A receptors in the inosine + PLA group, suggesting that inosine exerts its effects on regeneration and myelination through A2A receptor activation. However, further studies are needed to investigate which mechanisms are activated by A2A after injury and whether Schwann cells are involved.

## Data availability sattement

The original contributions presented in the study are included in the article/supplementary material, further inquiries can be directed to the corresponding author.
